# B-Mode and Contrast Enhanced Ultrasonography Features of Gastric Inflammatory and Neoplastic Diseases in Cats

**DOI:** 10.3390/ani10081444

**Published:** 2020-08-18

**Authors:** Francesco Simeoni, Rossella Terragni, Giuseppe Rubini, Roberto Tamburro, Francesca Del Signore, Ilaria Falerno, Giovanni Aste, Marco Russo, Giovanni Mastromatteo, Massimo Vignoli

**Affiliations:** 1 Faculty of Veterinary Medicine, University of Teramo, SP 18, 64100 Teramo, Italy; rtamburro@unite.it (R.T.); fdelsignore@unite.it (F.D.S.); ifalerno@unite.it (I.F.); giovanni.mastromatteo@gmail.com (G.M.); mvignoli@unite.it (M.V.); 2 Pet Care Veterinary Clinic, via Marzabotto ½ M-N, 40133 Bologna, Italy; terragni.rossella@gmail.com; 3 UltraVet Diagnostic, via Enrico Fermi 59, San Giovanni, 40017 Persiceto, Italy; giusepperubini@ymail.com; 4 Department of Veterinary Medicine and Animal Production, University of Naple, via Federico Delpino 1, 80137 Napoli, Italy; marco.russo@unina.it

**Keywords:** cats, gastric lymphoma, gastric inflammation, b-mode ultrasound, contrast enhanced ultrasound

## Abstract

**Simple Summary:**

Alimentary lymphoma is the most common neoplasm of the feline gastrointestinal tract. In most cases, standard ultrasonography may not be able to discriminate between a neoplastic and inflammatory infiltrate at this level. The aim of this prospective study is to establish the value of conventional ultrasound and contrast enhanced ultrasonography in describing specific features of normal, inflammatory, and neoplastic gastric diseases in feline species. A total of 29 cats were included in the study: six healthy cats as the control group; nine with gastric inflammation; three with low-grade alimentary lymphoma; 10 with high grade alimentary lymphoma. High-grade lymphoma usually appears as a severe wall thickening with absent layer definition, regional lymphadenopathy and local steatitis on a conventional ultrasound and shows a high-contrast uptake and a homogeneous enhancement with comb teeth-like vessels on contrast-enhanced ultrasound, while gastric inflammation and low-grade alimentary lymphoma showed a large overlap on both of those two ultrasonographic techniques. Diagnostic accuracy and cut-off value were calculated and found to be relevant for thickness (3.8 mm) for inflammation vs. low-grade lymphoma and “benign” vs. “malignant” as well as peak enhancement (34.87 dB) for “benign” vs. “malignant”. Thickness and peak enhancement can be useful parameters in the characterization of gastric infiltrates in cats.

**Abstract:**

Alimentary lymphoma (AL) is the most common malignancy of the feline gastrointestinal tract and may cause variable mild to severe alteration of the gastric wall on ultrasonography (US) that can be very similar to those caused by inflammation (INF). The aim of this prospective study is to establish the value of B-mode and contrast-enhanced US (CEUS) in describing specific features of normal, inflammatory, and neoplastic gastric diseases in feline species. B-mode US and CEUS of the stomach were performed in anesthetized cats with or without gastric disorders. Gastric wall qualitative and quantitative parameters were evaluated on B-mode US and CEUS examination. A total of 29 cats were included: six healthy (HEA) cats as the control group; nine INF; three low-grade lymphoma (LGAL); 10 high-grade lymphoma (HGAL). On B-mode US, there were significant differences in thickness, the wall’s layer definition and echogenicity between HGAL and all the other groups (<0.001). For CEUS, statistical differences between groups were found in the following: HGAL vs. HEA, HGAL vs. INF; HGAL vs. LGAL; INF vs. HEA. Diagnostic accuracy (AUC) and cut-off value were calculated and found to be significant for thickness (3.8 mm) for INF vs. LGAL (AUC > 0.70) and “benign” vs. “malignant” (AUC > 0.90) as well as peak enhancement (34.87 dB) for “benign” vs. “malignant” (AUC > 0.70). INF and LGAL showed an overlap of qualitative and quantitative parameters both on B-mode and CEUS, while HGAL usually appears as a severe wall thickening with absent layer definition, high-contrast uptake, a specific enhancement pattern, regional lymphadenopathy and local steatitis. Thickness and peak enhancement can be useful parameters in the characterization of gastric infiltrates in cats.

## 1. Introduction

Gastric tumours are rare in cats and, among them, alimentary lymphoma (AL) is the most commonly reported in this species [1,2,3,4,5,6]. AL can involve the gastrointestinal (GI) tract focally, multifocally or diffusely with the variable involvement of extra-gastrointestinal sites [4,5,7]. Classification includes several subtypes based on different histopathological characteristics including cell size (small or large), lymphocyte phenotype (T or B cell) and histological grade (low, intermediate or high) [4,5,7]. This classification recognizes two main forms of feline AL: low-grade alimentary lymphomas (LGAL), also called “mucosal lymphoma”, characterized by small T-cell infiltrated lymphocyte; a high-grade alimentary lymphomas (HGAL), also called “transmural lymphoma”, characterized by small or large B- or T-cell phenotypes [4,5].

In cats, clinical signs of AL are usually chronic and include weight loss, increased or decreased appetite, vomiting and diarrhea [5,7,8]. Abnormalities in bloodwork values may include: anemia and neutrophilia for complete cell blood count; hypoalbuminemia, panhypoproteinemia and increased liver enzymes, azotemia and bilirubin for serum biochemistry profile [5,7,8,9].

Ultrasonography (US) is the imaging modality of choice for the assessment of the canine and feline GI tract. Feline AL usually shows a circumferential, transmural thickening associated with diffuse loss of normal wall layering and reduced wall echogenicity, localized decreased motility and moderate regional lymphadenopathy [1,10,11]. Lymphoma can also show a focal hypoechoic eccentric mass [1,10]. However, US findings in the course of inflammatory diseases of the GI tract can be very similar to those of neoplastic infiltration: diffuse or multifocal mild wall thickening, loss of definition among wall layers and mesenteric lymphadenopathy [6,11,12]. Other non-neoplastic gastric lesions may have similar appearances to that of the wall’s neoplasia [13].

Contrast-enhanced ultrasonography (CEUS) is an imaging modality that could improve the diagnostic accuracy of standard US: the use of intravascular contrast medium (CM), like the second-generation ones, may improve the visualization of blood perfusion and microvascular architecture, allowing qualitative and quantitative vascular assessment of various organs in both physiological and pathological conditions in dogs and cats [14,15,16,17,18,19,20,21,22,23,24,25,26,27,28,29,30]. Furthermore, CEUS has proved to be an extremely safe and well-tolerated technique [31]. In veterinary medicine, several studies have described the usefulness of this modality in the characterization of inflammatory and neoplastic diseases [16,18,19,21,23,24,25,26,27,28,29,30,32]. However, regarding the stomach, only one study has been conducted to investigate the reliability of low-mechanical index imaging compered to Doppler flowmetry for the assessment of gastric mucosal blood flow in healthy dogs, demonstrating the feasibility of CEUS quantitative evaluation in this organ [33].

In human medicine, the CEUS technique has been used to perform qualitative and quantitative assessments of gastric wall perfusion in patients affected by gastritis and neoplastic disease [34,35]. One study reported that gastric cancer showed a diffuse enhancement without “comb teeth-like” vessels (parallel curvilinear structures representing arterial branching within the gastric wall), while this pattern was visible in most cases of gastritis; furthermore, the neoplastic wall showed delayed and lower peak enhancement compared to gastritis [34]. Another recent study evaluated standard B-mode US and CEUS in the characterization of malignant gastric tumours, reporting that the dynamics of the CM may be suggestive of the anatomopathological nature of the tumour: both adenocarcinomas and lymphomas showed a variable enhancement pattern followed by a delayed washout, while they differed in enhancement homogeneity; stromal tumours showed early arterial intense and homogenous enhancement followed by moderate washout in the venous phase [35].

The aim of our study is to establish the value of B-mode US and CEUS in describing specific features of normal, inflammatory, and neoplastic gastric diseases in feline species.

## 2. Materials and Methods

### 2.1. Animals

Conventional US and CEUS of the stomach were performed in cats with or without gastric disorders. This multicentric prospective study was approved by the Ethical Committee (Prot. 2016/0090753). The inclusion criteria were: (1) control group of healthy cats based on physical examination, serum chemistry profile, complete blood cell count and abdominal US which had to be anesthetized for planned castration or spaying, and absence of clinical and ultrasonographic signs compatible with gastric disorders confirmed by a follow-up of 12 months after the first B-mode and CEUS examination; (2) cats with gastric disorders enrolled based on clinical gastric related signs (chronic vomiting), serum chemistry profile, complete blood cell count, US abdominal examination, cytological or histological diagnosis of the gastric wall. All the patients included were fasted for at least 12 h before each study.

### 2.2. Anaesthesiology Protocol

A 20G catheter was inserted into the cephalic vein. The patients underwent the following anaesthetic protocol: sedation with butorphanol (Dolorex^®^, MSD A.H., 10 mg/mL), 0.3 mg/kg intramuscular, induction with propofol (Propovet^®^, Abbott Laboratories 10 mg/mL) intravenous, followed by tracheal intubation and maintenance with an Isoflurane/O_2_ mixture. Vital parameters such as heart rate, electrocardiogram, breathing rate, oxygen saturation and CO_2_ were monitored during the tests.

### 2.3. B-Mode US Examination

The patient, once induced, was prepared for US. The abdomen was clipped, and acoustic gel applied to the skin. The examinations were performed with the cats in right lateral recumbency, and a conventional US examination of the abdomen was performed. All the US were performed using a linear 7–12 MHz linear transducer on a dedicated machine (MyLab 70 XV and MyLab Twice, Esaote SpA, Genoa, Italy, Logiq S8, GE Healthcare Italy, Milan, Italy) with specific software for contrast agents. In cats with no evidence of gastric lesions on B-mode examination the CEUS study was performed at the level of the gastric body, while in patients with gastric lesions it was performed in that specific area.

For the B-mode evaluation, both quantitative and qualitative analysis were collected. The thickness of the gastric wall was measured, between the serosa and lumen interface, avoiding the rugal folds, in cats with uniform gastric wall thickness, and at the maximum thickness point in cats with evidence of lesions or masses. For gastric region appearance, the following B-mode qualitative features were evaluated: (1) layer definition, classified as normal, reduced or absent; (2) wall echogenicity, classified as normal or altered; (3) localization, classified as focal or diffuse; (4) regional lymph nodes, classified as normal or enlarged; (5) steatitis, classified as present or absent; (6) effusion, classified as present or absent.

### 2.4. CEUS Examination

The CM (Sonovue^®^, Bracco Imaging Italia srl, Milan, Italy) was injected by i.v., using a dose of 0.05 mL/kg followed by injection of 1 mL of bolus saline solution. A three-way valve was used to avoid any delay between the injection of CM and saline. Three contrast injections were performed for each case. The first injection was used to perfuse the blood vessels and to fine-tune the machine setting. The second and third injections were performed for gastric wall perfusion studies. Among the successive injections, to avoid artifacts, the remaining microbubbles were destroyed by modifying the acoustic power at the highest level and scanning the kidney, aorta, liver, and spleen for a few minutes. The basic technical parameters used were single focus, medium persistence, mechanical index 0.09 (40 kPa) and timer started at the end of the injection. For setting the gain, the starting point was a black/anechoic image, which represents the almost total suppression of the fundamental signal. The gain was optimized during the first injection to achieve a uniform image brightness. These settings have been repeated in the second and third injection. All studies were recorded for 90 s and digitized as a movie (clip) at a rate of 10 frames per second. The CEUS protocol reported above is similar to that already used and published in the veterinary literature [15,17,18,19,21,23].

**Figure 1:**
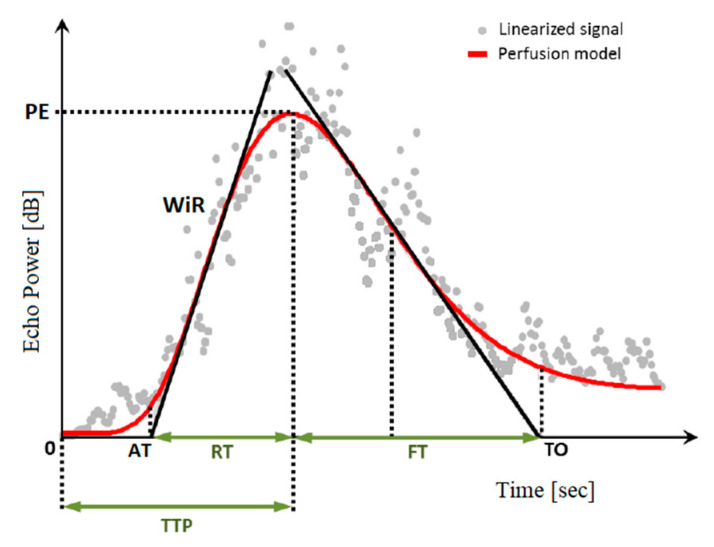
Examples of time–intensity curve explaining how peak enhancement (PE), wash-in rate (WiR), arrival time (AT), rising time (RT), time to peak (TTP), falling time (FT) and time to fall (TO) were calculated.

The clips were analysed using a dedicated software (VueBox^TM^, Bracco Imaging Italia srl, Milan, Italy) for quantitative analysis. A region of interest (ROI) was manually drawn by the same operator into the gastric wall: between the serous and the mucosal interface, in between the gastric folds, avoiding large vessels, in cats with no gastric lesions on conventional US; within lesions or masses, when possible, or into the pathological areas in animals suffering from gastric neoplasia. To optimize the CEUS quantitative analysis, the motion compensation software Veubox^TM^ was applied to minimize the effect of respiratory movements on the displacement of the ROI from the gastric wall. For each ROI, the software determined the average pixel intensity and created a time–intensity curve, which was subsequently analysed to calculate different blood flow quantitative parameters (Figure 1): blood volume, expressed by peak enhancement (PE) and Wash-in Rate (WiR) in decibels (dB); blood velocity, expressed by arrival time (AT), rising time (RT), time to peak (TTP), falling time (FT) and time to fall (TO), all in seconds (s). For each group of values and each study, the mean ± standard deviation (SD) were calculated and reported.

The qualitative assessment of enhancement patterns on CEUS clips was performed by the same operator in cases of a thickened gastric wall ≥6 mm only, since a thickness of less than 6 mm is too thin to perform an accurate analysis of the perfusion pattern. Moreover, even in human medicine, this type of analysis, performed in cases of gastritis and gastric neoplasia, has been reported only in ≥6-mm-thick gastric walls [34,35]. The enhancement patterns of thickened gastric walls considered are the same as those reported by Xue et al. in the human medicine literature [34]: (1) enhancement degree, considering visible normal liver parenchyma as the reference. The relative enhancement of the lesion at contrast arrival was classified as hyper-, iso-, or hypoenhancement; (2) homogeneity of contrast uptake, classified as homogeneous or heterogeneous enhancement; (3) comb teeth-like vessels, consisting of parallel curvilinear structures representing arterial branching within the gastric wall on CEUS, classified as present or absent [34]. These vessels can only be seen in the early arterial stage and usually last for about 1 s before being immersed in the diffused enhanced gastric wall. Because of this relatively short time window, CEUS clips have to be replayed frame by frame to determine their presence [34].

### 2.5. Characterization of Pathological Gastric Wall

To determine the nature of the gastric disease, in each of the patients, the gastric wall was sampled, guided by US or by endoscopy, and analysed by cytological or histological examination for characterization and classification. All gastric LGAL were confirmed by immunohistochemistry examinations.

### 2.6. Statistical Analysis

The statistical evaluation was performed using the MedCalc Statistical Software V.15 (Ostend, Belgium). Differences in the distribution of qualitative B-mode parameters among the different groups were tested with the Fisher’s exact method [36]. The differences in the distribution of the quantitative parameters in relation to the different groups were analysed using a one-way analysis of variance (ANOVA) for normally distributed data or with the Kruskal–Wallis test for non-normally distributed data. A value of *p* less than 0.05 was considered statistically significant for each test. The diagnostic accuracy of B-mode and CEUS quantitative parameters was assessed using the Receiver Operator Characteristic (ROC) curves between the groups. For each parameter, the cut-off point, area under the curve (AUC), sensibility (SE) and specificity (SP) were analysed. The AUC value was classified as low (0.5–0.7), moderate (0.7–0.9) or high (>0.9) as criteria of discrimination accuracy [37].

## 3. Results

### 3.1. Study Papulation

**Figure 2:**
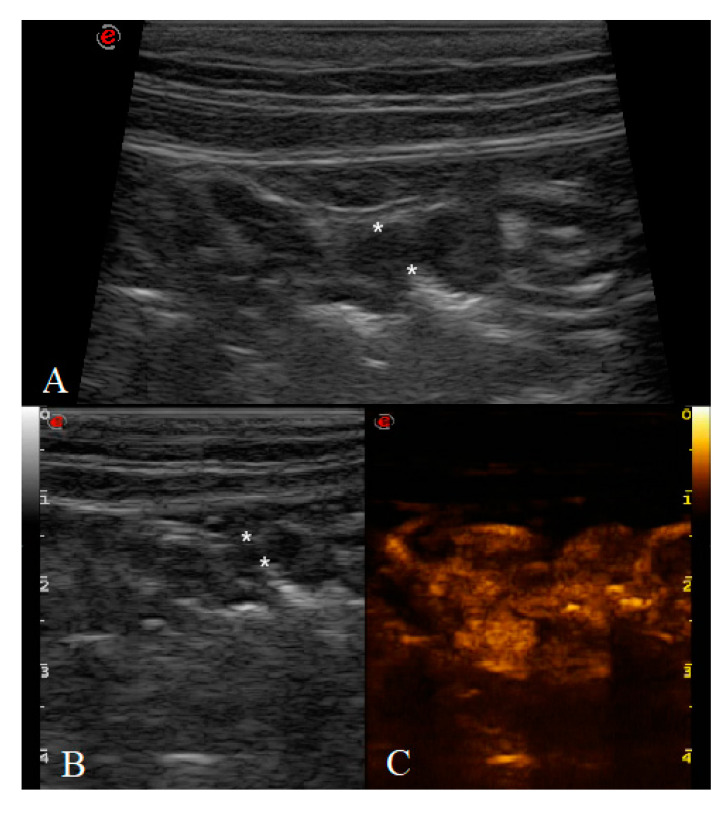
Cat affected by gastric low-grade lymphoma (LGAL) showing: (**A**) diffuse reduced layer definition associated with mild increases in wall thickness (3.8 mm between the asterisks) on B-mode examination; (**B**,**C**) B-mode (**B**) and contrast-enhanced ultrasonography (CEUS) (**C**) clip acquired using dual-mode visualization in order to better identify the gastric wall during CEUS examination.

**Figure 3:**
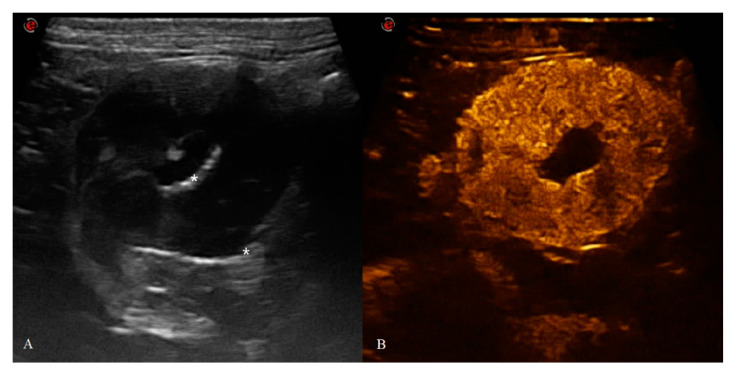
Feline gastric high-grade lymphoma (HGAL): (**A**) B-mode image of a severe and diffuse hypoechoic wall thickening of 18 mm (between the asterisks) with complete loss of normal wall layers; (**B**) CEUS examination of the same cat during the peak enhancement of time–intensity curve.

Between 2017 and 2020, a total of 31 cats underwent B-mode US and CEUS examination. Two cats were excluded because of the poor clip’s quality obtained from the CEUS examination and quantitative analysis could not be performed. All the gastric neoplasms were confirmed by cytological, histological and/or immunohistochemical diagnosis as lymphoma divided in LGAL and HGAL. No adverse effects were noted in any of the patients. A total of 29 cats were included in the study: six healthy (HEA) cats in the control group (mean age 2.08 ± 0.5 years), nine in the inflammatory (INF) group (mean age 6.4 ± 3.3 years), three in the LGAL group (mean age 12 ± 1 years) (Figure 2), and 10 in the HGAL group (mean age 9.4 ± 3.7 years) (Figure 3). All the HEA cats included in the control group were followed up on and checked 12 months after the first examination, confirming the absence of clinical and ultrasonographic signs of gastric disease.

### 3.2. Analysis of B-Mode Examination

**Figure 4:**
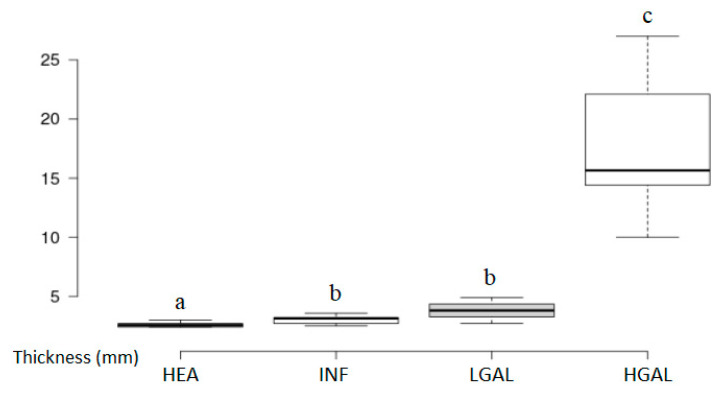
Box and whisker plot of the gastric wall thickness for healthy (HEA), inflammatory (INF), low-grade alimentary lymphoma (LGAL) and high-grade alimentary lymphoma (HGAL). ^a,b,c^ Values with different superscript letters differ significantly (*p* < 0.001).

The values and summary statistics of B-mode US measured gastric wall thickness with cytopathological classification are reported in Figure 4 and Table 1. The wall thickness in HGAL was significantly higher (<0.001) than the other groups, specifically 6.7 times the thickness of HEA, 5.5 times the thickness of INF, and 4.6 times the thickness of gastric walls affected by LGAL. Other statistical differences in thickness were found between HEA and INF (*p* = 0.021) and between HEA and LGAL (*p* = 0.028). No statistically significant difference was found between INF and LGAL (*p* = 0.074) in wall thickness.

**Figure 5:**
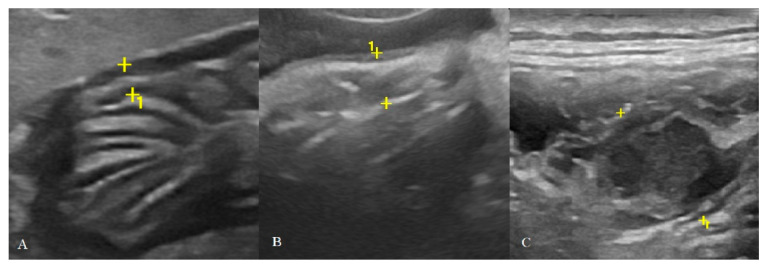
Classification of gastric wall layer definition on B-mode US: (**A**) normal: all the five layers are easily identified and thickness is normal (2.4 mm between the cursors in a HEA cat); (**B**) reduced: the identification of the layers is more difficult and, in some portions, it may not be evident, in addition to a mild diffuse wall thickening (3.6 mm between the cursors in a INF cat); (**C**) absent: it is not possible to recognize the normal wall stratification and layer definition is lost. Here, a focal transmural mass (16.5 mm between the cursors in a HGAL cat) with mixed echogenicity is also present.

**Figure 6:**
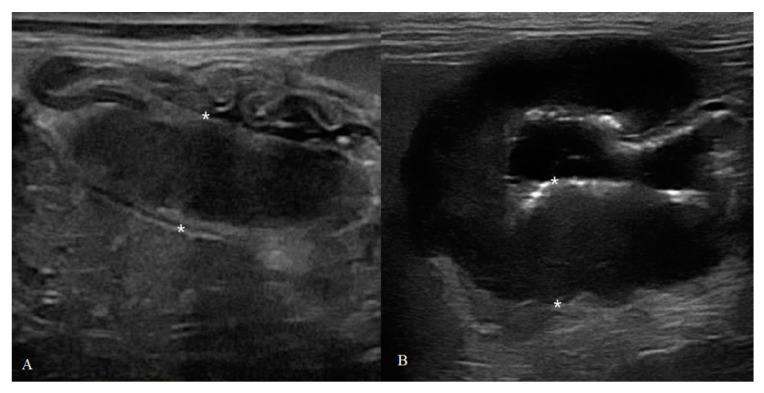
Different localization in gastric feline HGAL: (**A**) focal: a 7-year-old cat with a transmural mass (11.4 mm) with mixed echogenicity and loss of wall layers; (**B**) diffuse: severe hypoechoic thickening (20.8 mm) of gastric body with complete loss of normal layers in a cat 12-year-old cat.

Concerning the B-mode qualitative analysis, as reported in Table 2, there were significant differences in the wall’s layer definition (Figure 5) between HGAL and all the other groups (<0.001). The HEA cats had no alteration (0/6); gastric HGAL showed a complete loss of wall layer definition (10/10). The INF and LGAL showed a similar layer appearance with normal layering (6/9 INF and 2/3 LGAL) or diffuse reduced layering definition (3/9 and 1/3, respectively). Wall aspects were focal (4/10) or diffuse (6/10) in the HGAL group (Figure 6) and diffuse in inflammatory (9/9) and LGAL (3/3) groups. Echogenicity was significantly different between the groups (*p* < 0.001): altered in all HGAL lesions (10/10), with a hypoechoic aspect in 8/10 and only 2/10 with mixed echogenicity; for the INF group, only 2/9 cats showed an altered wall echogenicity, one with hyperechoic and one with hypoechoic aspects. Lymphadenopathy, characterized by rounded lymph nodes, was found in almost all HGAL (9/10), while it was not significant for LGAL (1/3) and INF (1/9) and absent in HEA (0/6). As for the perigastric region, steatitis was found in the majority of HGAL (8/10) and was absent in all other groups. Furthermore, regional effusion was found only in 2/10 HGAL.

### 3.3. Analysis of CEUS Examination

**Figure 7:**
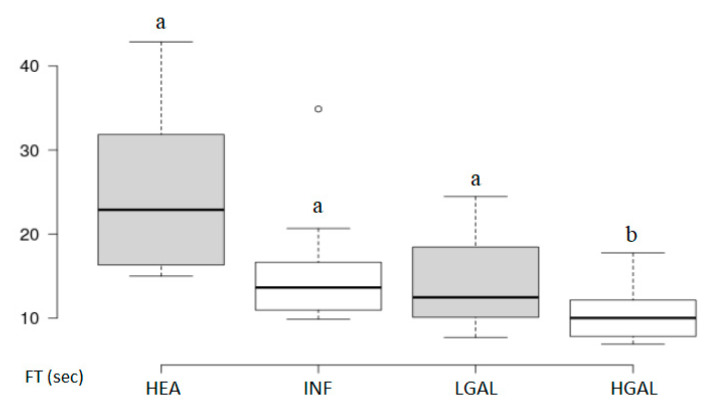
Box and whisker plot of the falling time (FT) for healthy (HEA), inflammatory (INF), low-grade alimentary lymphoma (LGAL) and high-grade alimentary lymphoma (HGAL). ^a,b^ Values with different superscript letters differ significantly (*p* < 0.05).

**Figure 8:**
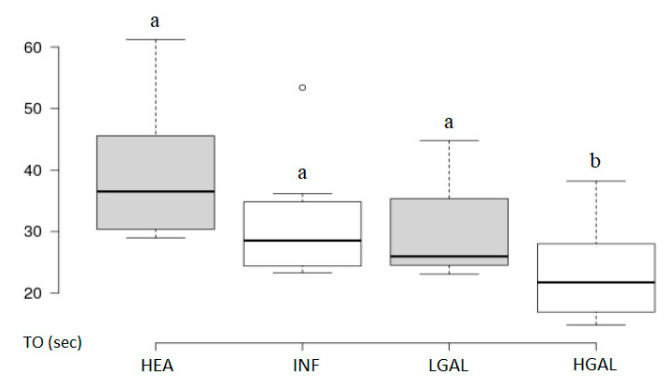
Box and whisker plot of the time to fall (TO) for healthy (HEA), inflammatory (INF), low-grade alimentary lymphoma (LGAL) and high-grade alimentary lymphoma (HGAL). ^a,b^ Values with different superscript letters differ significantly (*p* < 0.05).

**Figure 9:**
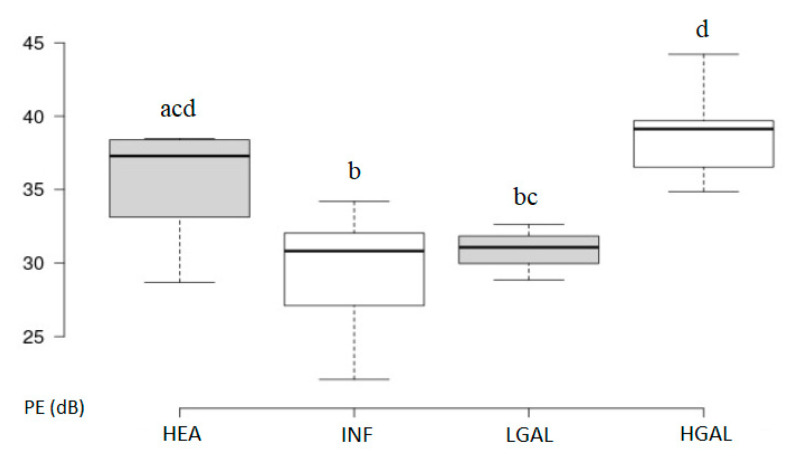
Box and whisker plot of the peak enhancement (PE) for healthy (HEA), inflammatory (INF), low-grade alimentary lymphoma (LGAL) and high-grade alimentary lymphoma (HGAL). ^a,b,c,d^ Values with different superscript letters differ significantly (*p* < 0.05).

**Figure 10:**
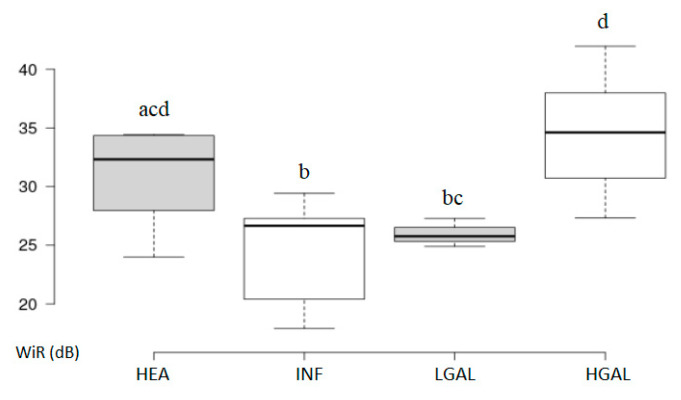
Box and whisker plot of the wash-in rate (WiR) for healthy (HEA), inflammatory (INF), low-grade alimentary lymphoma (LGAL) and high-grade alimentary lymphoma (HGAL). ^a,b,c,d^ Values with different superscript letters differ significantly (*p* < 0.05).

Summary statistics of the CEUS quantitative parameters for the different groups are reported in Table 3. Multiple comparison graphs of FT, TO PE and WiR, in which statistically significant differences were found between groups, are reported in Figure 7, Figure 8, Figure 9 and Figure 10, respectively. Since data collected for time and intensity values were normally distributed, the differences between groups were calculated by means with ANOVA.

CEUS quantitative values showed that HGAL has a higher contrast uptake compared to that of LGAL (*p* = 0.018 for PE and *p* = 0.01 for WiR) and INF (*p* < 0.001 both PE and WiR), but similar to that of HEA. HGAL differs from HEA for time values of FT (*p* = 0.001) and TO (*p* = 0.003), displaying a faster washout compared to that of the control group. No differences were evident between any other group for all the other time parameters. As for INF cats, they demonstrated a lower enhancement uptake compared to HEA, with significant differences for PE and WiR (*p* = 0.012 for both values) and similar to those of LGAL. It is interesting to observe how the contrast intensity values are similar between healthy stomachs and gastric walls compromised by severe tumor processes, suggesting that, despite the aggressiveness of the neoplastic process, there is no total vascular destruction, but rather a tendency towards proliferation.

**Figure 11:**
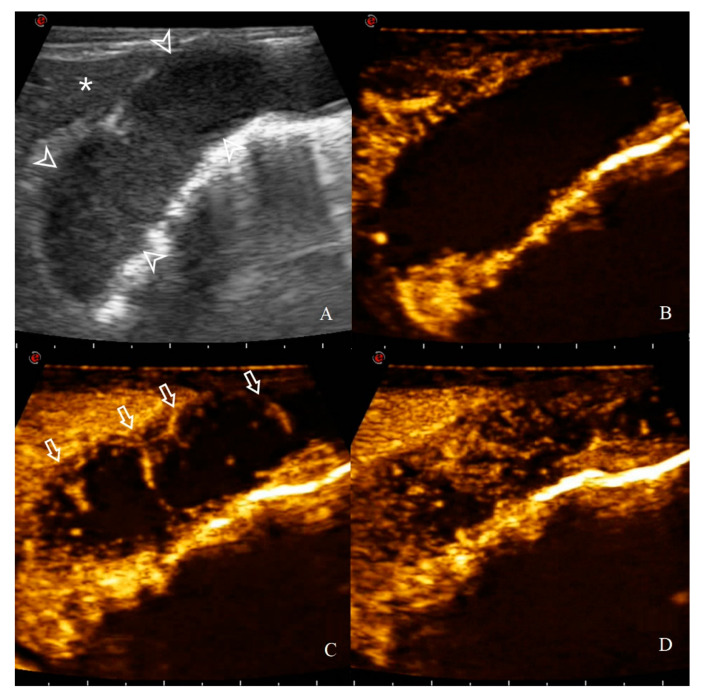
CEUS enhancement pattern in a 7-year-old cat with gastric HGAL: (**A**) before contrast medium (CM) injection (0 s), the gastric mass (14.8 mm between arrowheads) and the adjacent liver parenchyma (asterisk) are visualized; (**B**) after CM injection (8 s), uptake occurs first in the liver and the gastric mass is classified as hypo-enhancing; (**C**) when CM arrives at the lesion site (14 s), comb teeth-like vessels are present (arrows); (**D**) at peak enhancement time (18 s), arterial branching is not visible anymore and enhancement homogeneity is classified as heterogeneous.

CEUS qualitative assessments were evaluated only for the HGAL since all the cats with a gastric wall thickening ≥6 mm belong to this group. The enhancement patterns of the 10 HGAL on CEUS are reported in Table 4. HGAL showed a variable enhancement degree if compared to the adjacent liver parenchyma (5/10 hyper and 5/10 hypo/iso enhancement). Furthermore, lymphomas of this group were more likely to have a homogeneous enhancement (7/10) and the presence of comb teeth-like vessels (7/10) (Figure 11).

### 3.4. Diagnostic Accuracy in the Distinction between HEA, INF, LGAL and HGAL

Diagnostic accuracy using the ROC curve was evaluated by comparing the following groupings: (1) INF vs. LGAL, reported in Table 5; (2) “benign” (HEA + INF) vs. “malignant” (LGAL + HGAL) groups, as reported in Table 6. Only AT, TTP, PE, WiR and thickness were considered and the resulting AUC, SE, SP and cut-off point are reported in Table 4 and Table 5.

The thickness results were moderately accurate (AUC > 0.70) in discriminating INF from LGAL, with a cut-off point of 3.8 mm able to discriminate between this group with a sensitivity of 66.7%. Furthermore, the thickness results were highly accurate (AUC > 0.90) in discriminating “benign” from “malignant” also, with a cut-off of 3.8 mm and a sensitivity of 92%.

The PE results were moderately accurate (AUC > 0.70) in discriminating between “benign” and “malignant” groups with a cut-off point of 34.87 dB and a 77% sensitivity.

## 4. Discussion

The results of the present study reveal that the feline gastric wall has variable qualitative and quantitative characteristics in B-mode and CEUS during healthy, inflammatory, and neoplastic conditions. All cats in which a neoplastic infiltrate of the gastric wall was cytologically or histologically detected were affected by lymphoma (13/13), and this reflects what is reported in the veterinary literature, confirming this neoplasm as the most common gastric tumour [2,3,5,7].

B-mode US qualitative and quantitative characteristics of normal and pathological gastric walls have been reported in the veterinary literature throughout the years both in dogs and cats [1,10,11,38,39]. In our study, the average gastric interrugal thickness in healthy cats (mean 2.6 mm) was similar to that reported in the literature (around 2 mm) [1,40]. Focal or diffuse wall thickening with various echogenicity, associated with a loss of layer definition, are the main signs of HGAL [5,6,7,8,10,41,42]. However, LGAL is reported to have a similar B-mode US appearance to that of GI inflammatory diseases, causing no alteration or diffuse/multifocal mild wall thickening with lost/reduced/preserved layer definition and mesenteric lymphadenopathy [5,11,12,38].

In this study, data collected about B-mode qualitative and quantitative parameters partially confirm what is reported in the literature. The HGAL showed significantly higher thickness values than the other three groups, associated in all subjects with the complete loss of wall layers and alterations in echogenicity. Indeed, the majority of the cats showed a diffuse hypoechoic wall thickness or a focal hypoechoic transmural mass. Only one cat showed diffuse wall thickening with mixed echogenicity or focal mass with mixed echogenicity. These quantitative and qualitative characteristics match with those reported and described for HGAL by various authors throughout the years [1,9,10,11,41,42,43]. We found a statistically significant difference in wall thickness between HEA, INF and LGAL: although there were a certain number of overlapping data, LGAL group showed statistically significant differences in thickness to HEA but not with INF groups, with a slight increase in values from HEA to LGAL and intermediate values for the INF group. Concerning B-mode qualitative values, there were no statistically significant differences in terms of layer definition, localization, and echogenicity between these three groups, but only if compared to HGAL. These results agree with the literature for US findings in inflammatory and neoplastic disease of the small intestine both in dogs and cats [5,8,9,11,12,38,43,44]. These results indicate that, even for gastric involvement, inflammatory and low-grade lymphomatous infiltrates have similar US features, which are indistinguishable without an adequate histological or immunohistochemical characterization. Although a diffuse selective thickening of the muscularis layer was reported in cats with LGAL [9,41,45]; we found only 1/3 cats with reduced layer definition in LGAL group and only 2/9 with reduced layer definition in the INF group (Table 2) without selective thickening of the muscolaris layers. Furthermore, also considering regional lymphadenopathy, the presence of perigastric steatitis and effusion, we note that only the HGAL group showed clear alterations of these variables, confirming once again their local and distant aggressiveness.

All 31 cats that underwent CEUS examination showed no adverse reactions to the CM with stable respiratory and cardiovascular parameters during the examination time, confirming this technique as safe and well tolerated [31].

For CEUS quantitative parameters, the only statistical differences between groups were found in: HGAL vs. HEA (FT: *p* = 0.001; TO: *p* = 0.003), HGAL vs. INF (PE: *p* < 0.001; WiR: *p* < 0.001); HGAL vs. LGAL (PE: *p* = 0.018; WiR: *p* = 0.010) INF vs. HEA (PE: *p* = 0.012; WiR: *p* = 0.012). Other time and intensity parameters showed no significant differences between the groups. In our study population, HGAL showed higher intensity values compared to INF and LGAL, but similar to HEA, although HGAL differed from HEA for washout speed (FT and TO), which was faster for this neoplasm, with INF and LGAL having intermediate values compared to these and similar to each other. These quantitative findings seem to indicate very different vascular kinetics of feline gastric lymphomas from those shared for gastric malignancy reported in human medicine [34], where malignant lesions are reportedly characterized by lower values of contrast uptake (PE) and a delayed washout if compared to those of gastritis. However, the study performed by Xue et al. (2016) did not include any cases of gastric lymphoma, only adenocarcinomas, and this could explain the different vascular kinetics observed [34]. Furthermore, the study published by Neciu et al. (2019), performed on 30 patients, included three cases of gastric lymphoma in which, however, only a qualitative analysis of the CEUS examinations was performed [35].

Qualitative analysis on CEUS clips was performed by assessing the perfusion pattern only in HGAL, since the gastric wall thickness of the other groups was always <6 mm. HGAL shows a variable enhancement degree if compared to the adjacent liver (5/10 hyper and 5/10 hypo/iso enhancement). Furthermore, lymphomas of this group were more likely to have a homogeneous enhancement (7/10) and the presence of comb teeth-like vessels (7/10). In human medicine, enhancement patterns described for gastric lymphomas reported that these tumours are characterized by a variable uptake pattern, intense and heterogeneous enhancement, and a delayed washout [35]. However, in the mentioned study, the presence of comb teeth-like vessels was not considered, which was instead described by Xue et al. as typical of gastritis and not of gastric cancers [34]: this finding seems to further support, as already highlighted for the increased quantitative contrast uptake values (PE and WiR), that the vascular component of the neoplastic gastric wall, during feline HGAL, maintains increased and aberrant perfusion patterns.

To give useful indications for clinical practice, diagnostic accuracy was assessed, and thickness seems to be the most accurate diagnostic imaging finding in differentiating a neoplastic (LGAL and HGAL) from a non-neoplastic gastric wall (HEA and INF). Considering only the INF and LGAL groups, the ROC curve showed a low diagnostic value for the parameters considered, except for the thickness, which is moderately accurate (AUC > 0.70) in separating cats with inflammatory lesions from those with LGAL: a gastric wall thickening higher than 3.8 mm may indicate LGAL rather than a gastritis. On the other hand, considering the whole series (classifying the HEA and INF as “benign” and the LGAL plus HGAL as “malignant”), the PE and WiR parameters are moderately accurate, while the thickness is highly accurate. Thickness can therefore be considered as a highly diagnostic parameter in identifying animals with neoplastic infiltrates (low or high grade). Cut-off values for thickness (>3.8 mm) and PE (>34.87 dB) can both help to discriminate between a gastric lymphoma and a healthy or inflammatory gastric wall with high and moderate diagnostic values, respectively. This data shows that the evaluation of these two combined B-mode and CEUS quantitative parameters can increase the diagnostic accuracy in these pathological conditions, especially if only mild alterations are present in B-mode qualitative evaluation. The potential limitations of this study include the difficulty in gastric wall measurement for the CEUS parameter evaluation program, especially if no significant thickenings or masses are present, due to the limited space in which the ROI can be drawn within the gastric wall; furthermore, gas reverberation artifacts and breathing movement may alter the measurements at the ROI site. Other study limitations relate to the animals included: the lack of cyto- or histopathological examination of the gastric wall for the HEA control group, in which, however, the absence of clinical, laboratory and ultrasonographic signs compatible with gastric disease was confirmed both at the first check and at the follow-up after 12 months, while the reduced number of cats affected by LGAL was collected.

## 5. Conclusions

The results of this study demonstrate how gastric inflammation and lymphoma in cats can show different B-mode and CEUS features. Cats with gastric INF and LGAL show a large overlap of both qualitative and quantitative parameters, both in B-mode and CEUS methods, once again confirming the difficulty in the discrimination between these two conditions. Instead, HGAL showed a well-defined B-mode and CEUS characteristics sufficient to differentiate them from other physiological and pathological conditions. Future studies could focus on further evaluations of the feline GI tract in the discrimination between LGAL and inflammatory conditions using other US techniques such as elastosonography.

## Figures and Tables

**Table 1:** Wall thickness B-mode evaluation for healthy (HEA), inflammatory (INF), low-grade alimentary lymphoma (LGAL) and high-grade alimentary lymphoma (HGAL) reported as means with standard deviations (±SD).

WALL THICKNESS (mm)	HEA	INF	LGAL	HGAL	*p*-VALUE
***Mean***	2.6 ^a^	3.02 ^b^	3.8 ^b^	17.6 ^c^	<0.001
***±SD***	0.22	0.35	1.1	5.7	
***Range***	2.4–3	2.5–3.6	2.7–4.9	10–27	

^a,b,c^ Values with different superscript letters differ significantly (*p* < 0.001).

**Table 2:** Number of cases (*n*) and percentage (%) showing qualitative features at B-mode ultrasound for healthy (HEA), inflammatory (INF), low-grade alimentary lymphoma (LGAL) and high-grade alimentary lymphoma (HGAL).

B-MODE QUALITATIVE VARIABLES	HEA (n = 6) %	INF (n = 9) %	LGAL (n = 3) %	HGAL (n = 10) %	*p*-Value
**Layer definition**					<0.001
*Normal*	100 ^a^	77.7 ^a^	66.6 ^a^	0 ^b^	
*Reduced*	0 ^a^	22.2 ^a^	33.3 ^a^	0 ^b^	
*Absent*	0 ^a^	0 ^a^	0 ^a^	100 ^b^	
**Localization**					0.08
*Focal*	NA	0	0	40	
*Diffuse*	NA	100	100	60	
**Echogenicity**					<0.001
*Normal*	100 ^a^	77.7 ^a^	100 ^a^	0 ^b^	
*Altered*	0 ^a^	22.2 ^a^	0 ^a^	100 ^b^	
**Regional limph nodes**					
*Normal*	100	88.8	66.6	10	
*Enlarged*	0	11.1	33.3	90	
**Steatitis**					
*Present*	0	0	0	80	
*Absent*	100	100	100	20	
**Effusion**					
*Present*	0	0	0	20	
*Absent*	100	100	100	80	

^a,b^ Values with different superscript letters differ significantly (*p* < 0.001). Not available (NA).

**Table 3:** Quantitative parameters reported as mean values of CEUS examination for healthy (HEA), inflammatory (INF), low-grade alimentary lymphoma (LGAL) and high-grade alimentary lymphoma (HGAL).

CEUS QUANTITATIVE FEATURES	HEA (n = 6)	INF (n = 9)	LGAL (n = 3)	HGAL (n = 10)	SEM	*p*-Value
**AT (*s*)**	8.96	9.83	11.06	8.28	2.54	0.44
**RT (*s*)**	5.59	5.31	5.34	4.21	1.93	0.50
**TTP (*s*)**	14.55	15.15	16.41	12.49	2.96	0.34
**FT (*s*)**	25.30 ^a^	15.99 ^a^	14.87 ^a^	10.44 ^b^	4,99	0.007
**TO (*s*)**	39.85 ^a^	31.14 ^a^	31.28 ^a^	22.94 ^b^	6,81	0.021
**PE (*dB*)**	35.53 ^a c d^	29.48 ^b^	30.85 ^b c^	38.99 ^d^	1.59	<0.001
**WiR (*dB*)**	30.89 ^a c d^	24.29 ^b^	25.98 ^b c^	34.91 ^d^	1.74	<0.001

^a,b,c,d^ Values with different superscript letters differ significantly (*p* < 0.05). Standard error of the mean (SEM).

**Table 4:** Number of cases (*n*) and percentage (%) showing qualitative variables (enhancement pattern) on CEUS for high-grade alimentary lymphoma (HGAL).

CEUS QUALITATIVE VARIABLES	HGAL % (n = 10)
***Enhancement degree***	
*Hyperenhancement*	50 (5/10)
*Hypo/Isoenhancement*	50 (5/10)
***Enhancement homogeneity***	
*Homogeneous*	70 (7/10)
*Heterogeneous*	30 (3/10)
***Comb teeth-like vessels***	
*Present*	70 (7/10)
*Absent*	30 (3/10)

**Table 5:** Parameters calculated from Receiver Operator Characteristic (ROC) curves of B-mode and CEUS quantitative values for discrimination between low-grade alimentary lymphoma (LGAL) and inflammation (INF).

	LGAL	INF		
*CATS* (*n*)	3	9		
	AUC	CUT-OFF	SE, %	SP, %
*AT* (*s*)	0.65	11.2	66.67	77.8
*RT* (*s*)	0.37	9.14	33.33	88.89
*TTP* (*s*)	0.63	18.27	66.67	77.78
*PE* (*dB*)	0.56	28.86	100	44.44
*WiR* (*dB*)	0.54	24.9	100	44.44
*Thickness* (*mm*)	0.76	3.8	66.67	100

**Table 6:** Parameters calculated from Receiver Operator Characteristics (ROC) curves of B-mode and CEUS quantitative values for discrimination between “benign” and “malignant” groups.

	Benign	Malignant		
*CATS* (*n*)	15	13		
	AUC	CUT-OFF	SE, %	SP, %
*AT* (*s*)	0.58	6.6	46.15	93.33
*RT* (*s*)	0.72	4.25	69.23	80
*TTP* (*s*)	0.62	11.55	53.85	93.33
*PE* (*dB*)	0.79	34.87	76.92	73.33
*WiR* (*dB*)	0.77	34.56	46.15	100
*Thickness* (*mm*)	0.96	3.8	92.31	100
